# Supplementary material for: Systemic and Mucosal Immune Responses Induced by Adenoviral-Vectored Consensus H5 Influenza A Vaccines in Mice and Swine
Source: Vaccines (Basel). 2025 Aug 30;13(9):928. doi: 10.3390/vaccines13090928 (PMC12474482; doi:10.3390/vaccines13090928)
Supplement: Supplementary file 1 [file vaccines-13-00928-s001.zip › vaccines-3831927-supplementary.pdf]

Table S1: Percentage(%) of Amino Acid Identity of the Hemagglutinin (HA) ptotein

| **Supplemental Table S1** | **Percentage (%) of Amino Acid Identity of the Hemagglutinin (HA) Protein** | | | | | | | |
|---|---|---|---|---|---|---|---|---|
| | **H5CC** | **A/Vietnam/1204/2004 (Vietnam/2004)** | **A/bar-headed goose/Qinghai/A/2005 (Goose/2005)** | **A/Japanese white-eye/Hong Kong/1038/2006 (White-eye/2006)** | **A/northern pintail/Washington/40961/2014 (Northern pintail/2014)** | **A/snow goose/Missouri/CC15-84A/2015 (Snow goose/2015)** | **A/bald eagle/Florida/W22-134-OP/2022 (Bald eagle/2022)** | **A/bovine/Ohio/B24OSU-439/2024 (Bovine/2024)** |
| **H5CC** | | 99.3 | 92.8 | 96.5 | 92.6 | 92.8 | 92.3 | 92.3 |
| **A/Vietnam/1204/2004 (Vietnam/2004)** | 99.3 | | 96.8 | 95.9 | 92.1 | 92.3 | 91.7 | 91.7 |
| **A/bar-headed goose/Qinghai/A/2005 (Goose/2005)** | 92.8 | 96.8 | | 96.5 | 92.3 | 92.1 | 91.7 | 91.7 |
| **A/Japanese white-eye/Hong Kong/1038/2006 (White-eye/2006)** | 96.5 | 95.9 | 96.5 | | 93.3 | 93.1 | 93.3 | 93.3 |
| **A/northern pintail/Washington/40961/2014 (Northern pintail/2014)** | 92.6 | 92.1 | 92.3 | 93.3 | | 99.6 | 95.6 | 95.4 |
| **A/snow goose/Missouri/CC15-84A/2015 (Snow goose/2015)** | 92.8 | 92.3 | 92.1 | 93.1 | 99.6 | | 95.4 | 95.2 |
| **A/bald eagle/Florida/W22-134-OP/2022 (Bald eagle/2022)** | 92.3 | 91.7 | 91.7 | 93.3 | 95.6 | 95.4 | | 99.6 |
| **A/bovine/Ohio/B24OSU-439/2024 (Bovine/2024)** | 92.3 | 91.7 | 91.7 | 93.3 | 95.4 | 95.2 | 99.6 | |
